# Supplementary material for: Nano-org, a functional resource for single-molecule localisation microscopy data
Source: Nat Commun. 2025 Sep 30;16:8674. doi: 10.1038/s41467-025-63674-x (PMC12484560; doi:10.1038/s41467-025-63674-x)
Supplement: Supplementary file 1 — Supplementary Information File [file 41467_2025_63674_MOESM1_ESM.pdf]

Supplementary Figures

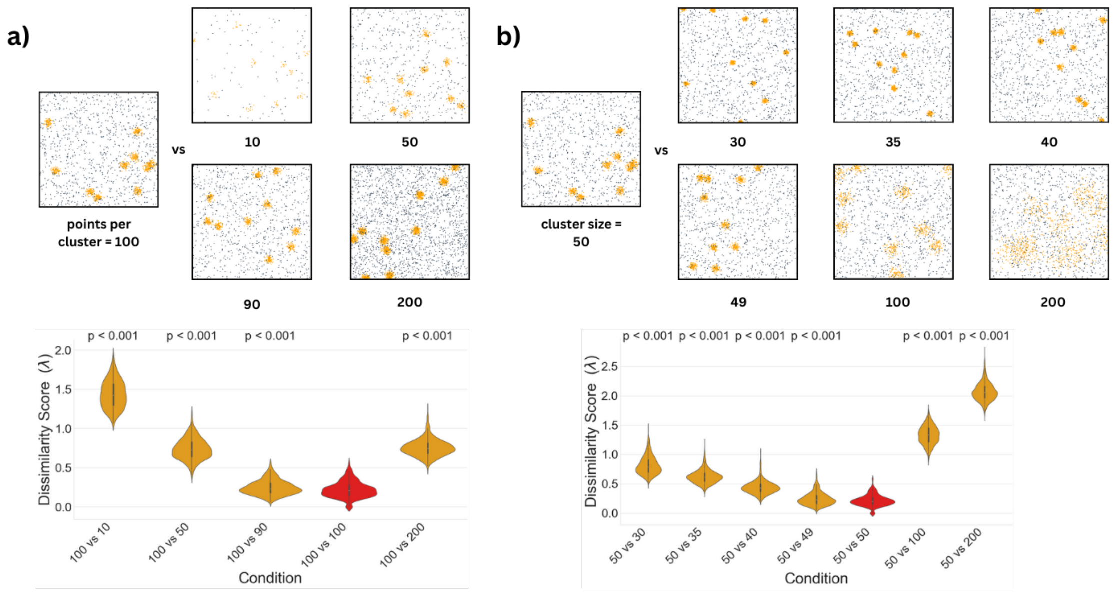

Figure S1: Dissimilarity scores between simulated Gaussian clusters. **a)** Example ROIs for each Gaussian cluster simulation with different numbers of points per cluster, with dissimilarity scores comparing 100 points per cluster with all other conditions (30 ROIs per condition). **b)** Example ROIs for each Gaussian cluster simulation with different cluster sizes, with dissimilarity scores comparing clusters of size 50 nm with itself and all other conditions (30 ROIs per condition).

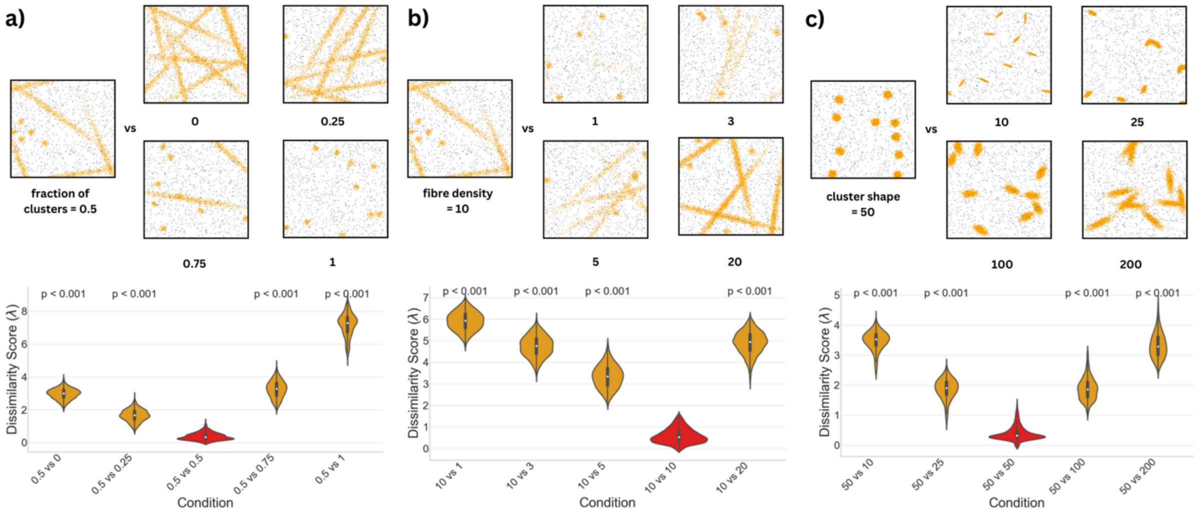

Figure S2: Dissimilarity scores between simulated alternative structures. a) Example ROIs from simulations in which each region contains 10 structures (shown in yellow), with the fraction of those structures defined as Gaussian clusters varying from 0 to 1. The remaining structures are modelled as fibrous features. Dissimilarity scores are shown comparing a condition with 50% clusters (fraction = 0.5) to all other ratios (30 ROIs per condition). **b)** Example ROIs from simulations in which each region contains 5 fibrous structures and 5 clusters. Fibre density refers to the number of localisations per fibrous structure relative to the cluster structures, which are held constant. A fibre density of 1 indicates equal localisation density between fibre and cluster structures, while a density of 10 means each fibre contains ten times as many localisations as each cluster. Dissimilarity scores are shown comparing a fibre density of 10 to all other conditions (30 ROIs per condition). **c)** Example ROIs from simulations in which cluster shape is varied by generating elliptical structures. One axis is fixed at 50 nm, while the other axis is adjusted from 10 to 200 nm, altering the ellipticity of the clusters. A shape parameter of 50 corresponds to a circular Gaussian cluster where the major and minor axes are equal. Dissimilarity scores are shown comparing this reference condition (minor axis = major axis = 50 nm) to all other elliptical shapes (30 ROIs per condition).

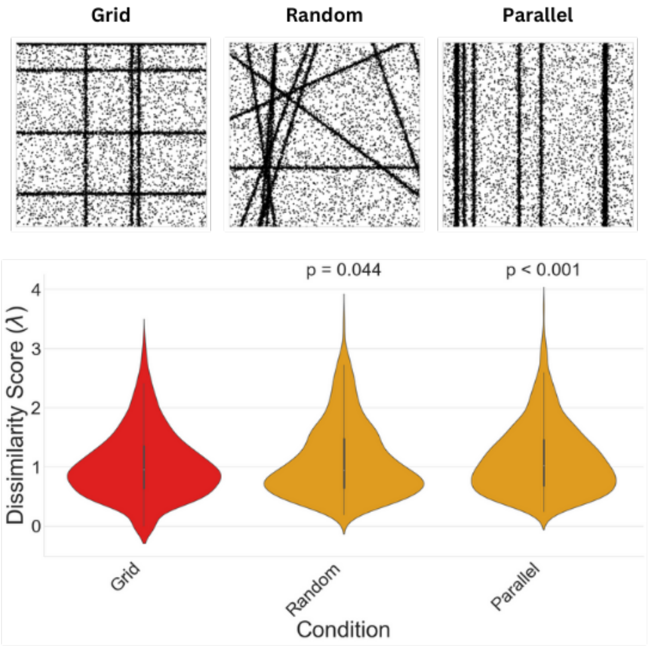

Figure S3: Dissimilarity scores between simulated fibres. Fibres are arranged on a grid, randomly, or parallel to each other. Dissimilarity scores between fibres arranged on a grid are compared to themselves and with randomly oriented and parallel fibres (30 ROIs per condition).

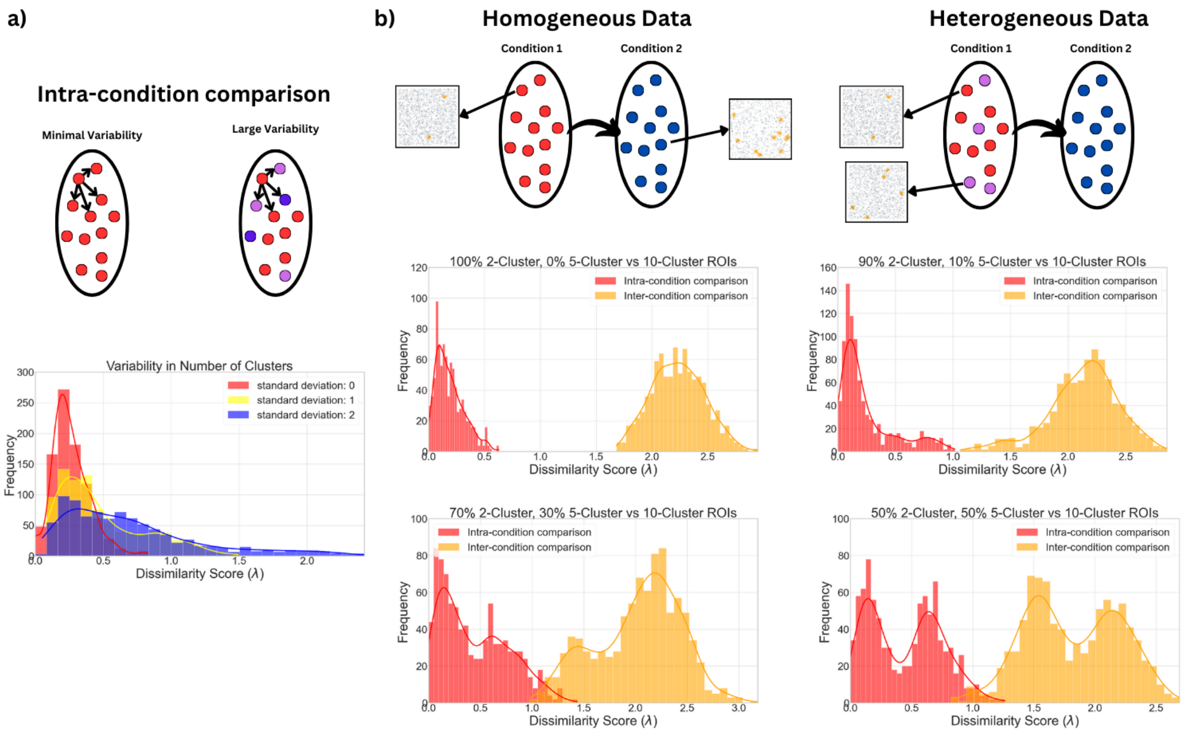

Figure S4: Self-similarity scores reveal intra-condition variability and the presence of subpopulations. The self-similarity score is used to assess intra-condition variability, testing how consistent molecular distributions are within a dataset. **a)** Intra-condition comparisons are performed by computing dissimilarity scores between individual regions of interest (ROIs) and all other ROIs within the same condition. In the schematic, each oval represents a condition with each circle within the oval representing a single ROI within that condition, and arrows indicate the comparison of one ROI against all others. In the left condition, all ROIs have identical characteristics (e.g. same number of clusters, shown in red), resulting in minimal variability. In the right condition, ROIs vary in their properties (e.g. cluster number), illustrated by circles of different colours, leading to higher intra-condition dissimilarity. The plot below shows distributions of intra-condition dissimilarity scores for datasets where the number of clusters per ROI is fixed (red), or drawn from a normal distribution with a mean of 10 and a standard deviation of 1 (yellow) or 2 (blue) (30 ROIs per condition). Greater variability in the number of clusters leads to broader score distributions, indicating increased heterogeneity**. b)** The similarity histograms demonstrate how variability within a dataset affects the distribution of similarity scores. In this case, one condition contains two distinct subpopulations of ROIs—each ROI either contains 2 clusters or 5 clusters. The percentage of each subpopulation is indicated. The self-similarity histogram for this condition shows two distinct peaks, corresponding to the two subpopulations, highlighting the dataset's heterogeneity. Inter-condition similarity is then computed by comparing this mixed population to a homogeneous population containing 10 clusters per ROI (30 ROIs per condition). The histograms illustrate how variability within a dataset influences the similarity scores, with greater heterogeneity leading to broader distributions and multi-modal peaks.

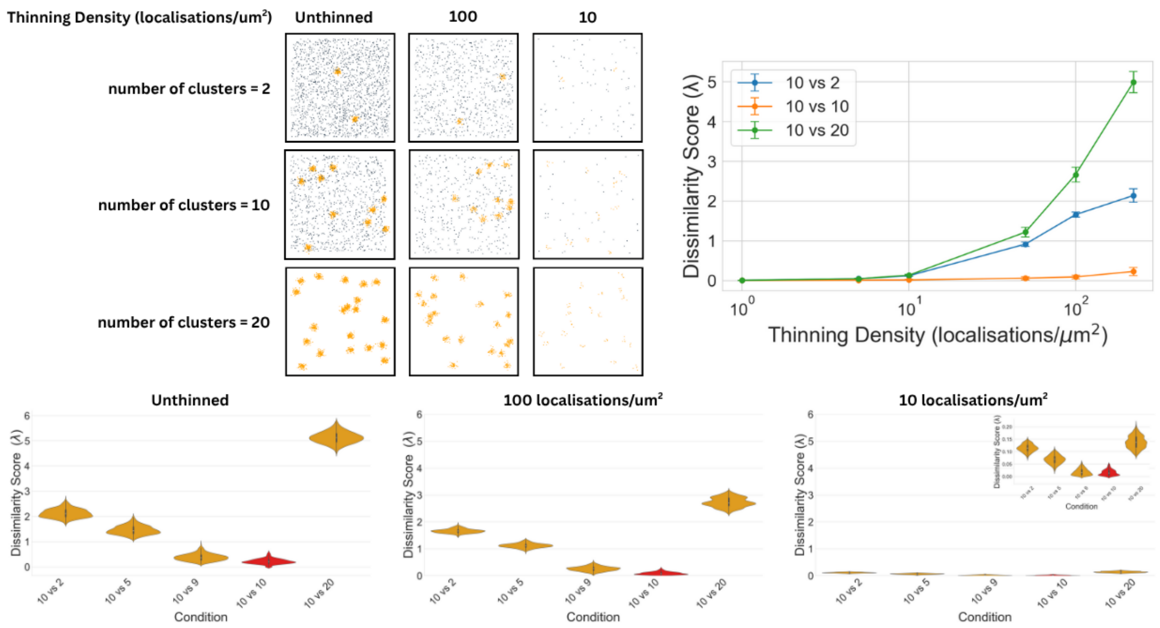

Figure S5: Effect of thinning on dissimilarity scores between simulated clustering patterns. Simulated 3×3 μm² ROIs with 2, 10, or 20 clusters are compared to a reference set with 10 clusters per ROI (30 ROIs per condition). Each ROI is progressively thinned to target densities of 100 and 10 localisations/μm². The line plot shows the mean dissimilarity score (λ) between the 10-cluster reference and datasets with 2, 10, and 20 clusters across thinning densities. Dissimilarity increases with more extreme differences in clustering but becomes less pronounced as density decreases. Violin plots show the distribution of dissimilarity scores for comparisons against the 10-cluster dataset at three thinning densities: unthinned, 100, and 10 localisations/μm². Differences between clustering conditions become harder to distinguish at lower densities, indicating reduced sensitivity of the similarity metric under sparse localisation conditions.

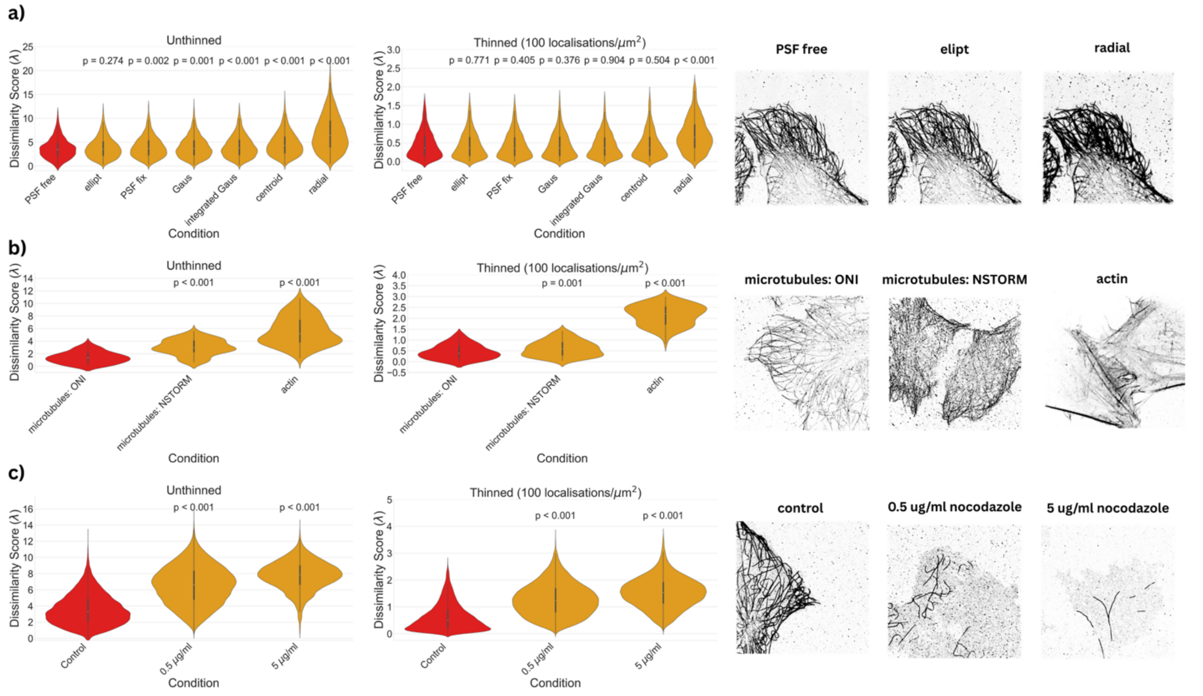

Figure S6: Dissimilarity scores between experimental datasets. Violin plots show pairwise dissimilarity scores (λ) between datasets, with example single-cell localisations shown on the right. **a)** Comparison of the same dataset (microtubules in COS-7 cells labelled with Alexa Fluor 647) processed using various fitting algorithms implemented in SMAP or ThunderSTORM (43 ROIs in each condition). Most algorithms yield reconstructions that are visually and quantitatively similar to the reference (PSF-free in SMAP), with 'ellipt' (SMAP) producing the closest match. The 'radial' fitting method in ThunderSTORM deviates most. **b)** Comparison of the same biological sample (microtubules in COS-7 cells labelled with Alexa Fluor 647) imaged on two different microscopes (ONI (10 ROIs) and N-STORM (11 ROIs)), alongside a distinct dataset of actin in HeLa cells labelled with Alexa Fluor 488 (18 ROIs) and acquired by a different lab. While some dissimilarity exists between reconstructions of the same sample on different platforms, the scores are notably lower than those obtained from a biologically distinct sample. **c)** Comparison of microtubules in COS-7 cells treated with increasing concentrations of nocodazole (0 (209 ROIs), 0.5 (390 ROIs), and 5 μg/ml (234 ROIs)). Cells treated with 0.5 μg/ml nocodazole show some differences compared to untreated controls, and cells treated with 5 μg/ml nocodazole exhibit markedly greater dissimilarity, reflecting substantial disruption of microtubule structure.
