# Supplementary material for: Nano-org, a functional resource for single-molecule localisation microscopy data
Source: Nat Commun. 2025 Sep 30;16:8674. doi: 10.1038/s41467-025-63674-x (PMC12484560; doi:10.1038/s41467-025-63674-x)
Supplement: Supplementary file 2 — Reporting summary [file 41467_2025_63674_MOESM2_ESM.pdf]

nature research

Corresponding author(s): Dylan M. Owen

Last updated by author(s): 08/20/2025

Reporting Summary

Nature Research wishes to improve the reproducibility of the work that we publish. This form provides structure for consistency and transparency in reporting. For further information on Nature Research policies, see our Editorial Policies and the Editorial Policy Checklist.

Statistics

For all statistical analyses, confirm that the following items are present in the figure legend, table legend, main text, or Methods section.

n/a	Confirmed	
☐	☒	The exact sample size (*n*) for each experimental group/condition, given as a discrete number and unit of measurement
☐	☒	A statement on whether measurements were taken from distinct samples or whether the same sample was measured repeatedly
☐	☒	The statistical test(s) used AND whether they are one- or two-sided<br>*Only common tests should be described solely by name; describe more complex techniques in the Methods section.*
☒	☐	A description of all covariates tested
☒	☐	A description of any assumptions or corrections, such as tests of normality and adjustment for multiple comparisons
☐	☒	A full description of the statistical parameters including central tendency (e.g. means) or other basic estimates (e.g. regression coefficient) AND variation (e.g. standard deviation) or associated estimates of uncertainty (e.g. confidence intervals)
☐	☒	For null hypothesis testing, the test statistic (e.g. *F*, *t*, *r*) with confidence intervals, effect sizes, degrees of freedom and *P* value noted<br>*Give P values as exact values whenever suitable.*
☒	☐	For Bayesian analysis, information on the choice of priors and Markov chain Monte Carlo settings
☒	☐	For hierarchical and complex designs, identification of the appropriate level for tests and full reporting of outcomes
☒	☐	Estimates of effect sizes (e.g. Cohen's *d*, Pearson's *r*), indicating how they were calculated

Our web collection on statistics for biologists contains articles on many of the points above.

Software and code

Policy information about availability of computer code

Data collection	ONI Nanoimager software v1.19.4,<br>SMAP, version 1.0.0.0 (1)<br>FIJI (ImageJ) v2.14.0/1.54f, ThunderSTORM ImageJ plugin (dev-2018-04-13-pSMLM)
Data analysis	The implementation of the website and database is available at https://gitlab.bham.ac.uk/owendz-protein-databank/nano-org-website<br><br>The core analysis functionality and algorithms used by nano-org are implemented as a stand-alone python package which is available at https://gitlab.bham.ac.uk/owendz-protein-databank/smlm-analysis<br><br>All Python scripts used to produce simulated data and violin plots in figures and supplementary figures are available at https://gitlab.bham.ac.uk/owendz-protein-databank/nano-org-similarity-scoring<br><br>Anaconda Navigator 2.6.6<br>Python 3.11.5<br>Spyder 6.0.1 (conda)

For manuscripts utilizing custom algorithms or software that are central to the research but not yet described in published literature, software must be made available to editors and reviewers. We strongly encourage code deposition in a community repository (e.g. GitHub). See the Nature Research guidelines for submitting code & software for further information.

Data

Policy information about availability of data

All manuscripts must include a data availability statement. This statement should provide the following information, where applicable:

- Accession codes, unique identifiers, or web links for publicly available datasets
- A list of figures that have associated raw data
- A description of any restrictions on data availability

All experimental data is stored and available for download on https://nano-org.bham.ac.uk
Simulated data can be found in https://gitlab.bham.ac.uk/owendz-protein-databank/nano-org-similarity-scoring

Field-specific reporting

Please select the one below that is the best fit for your research. If you are not sure, read the appropriate sections before making your selection.

☒ Life sciences ☐ Behavioural & social sciences ☐ Ecological, evolutionary & environmental sciences

For a reference copy of the document with all sections, see nature.com/documents/nr-reporting-summary-flat.pdf

Life sciences study design

All studies must disclose on these points even when the disclosure is negative.

Sample size	No sample size calculation was performed. Sample size was determined to approximately match typical sample sizes found in the literature
Data exclusions	None
Replication	3 technical replicates were performed, data was consistent between replicates
Randomization	No randomization as no comparison of experimental data sets is made
Blinding	No blinding as no comparison of experimental data sets is made

Reporting for specific materials, systems and methods

We require information from authors about some types of materials, experimental systems and methods used in many studies. Here, indicate whether each material, system or method listed is relevant to your study. If you are not sure if a list item applies to your research, read the appropriate section before selecting a response.

Materials & experimental systems

n/a	Involved in the study	
☐	☒	Antibodies
☐	☒	Eukaryotic cell lines
☒	☐	Palaeontology and archaeology
☒	☐	Animals and other organisms
☒	☐	Human research participants
☒	☐	Clinical data
☒	☐	Dual use research of concern

Methods

n/a	Involved in the study	
☒	☐	ChIP-seq
☒	☐	Flow cytometry
☒	☐	MRI-based neuroimaging

Antibodies

Antibodies used	mouse monoclonal beta-tubulin IgG3 primary antibody (200ug/mL, Santa Cruz Biotechnology, sc-166729)<br>Alexa FluorTM 647-conjugated goat anti-mouse IgG secondary antibody (2mg/mL, Life Technologies, A21235)
Validation	sc-166729 - https://www.scbt.com/p/beta-tubulin-antibody-f-1?srsltid=AfmBOopAJHg5X8lHkfYnKEICSFfhgKq3lNBFH7nP8RUf6UC5CQloAqZk<br><br>A21235 - https://www.thermofisher.com/antibody/product/Goat-anti-Mouse-IgG-H-L-Cross-Adsorbed-Secondary-Antibody-Polyclonal/A-21235

Eukaryotic cell lines

Policy information about cell lines

Cell line source(s)	COS-7 cells gifted from Professor Dirk-Peter Herten
Authentication	Not authenticated
Mycoplasma contamination	Not tested
Commonly misidentified lines (See ICLAC register)	None
